# Combined bulked segregant sequencing and traditional linkage analysis for identification of candidate gene for purple leaf sheath in maize

**DOI:** 10.1371/journal.pone.0190670

**Published:** 2018-01-05

**Authors:** Pengcheng Li, Cancan Du, Yingying Zhang, Shuangyi Yin, Enying Zhang, Huimin Fang, Dezhou Lin, Chenwu Xu, Zefeng Yang

**Affiliations:** 1 Jiangsu Key Laboratory of Crop Genetics and Physiology/Co-Innovation Center for Modern Production Technology of Grain Crops, Key Laboratory of Plant Functional Genomics of the Ministry of Education, Yangzhou University, Yangzhou, China; 2 College of Agronomy and Plant Protection, Qingdao Agricultural University, Qingdao, China; National Institute for Plant Genome Research, INDIA

## Abstract

Anthocyanin accumulation in various maize tissues plays important roles in plant growth and development. In addition, some color-related traits can be used as morphological markers in conventional maize breeding processes and purity identification of hybrid seeds. Here, we noticed that the leaf sheath color was controlled by a dominant gene, because purple (PSH) and green leaf sheaths (GSH) were separated at a ratio of 3:1 in an F_2_ population. To map the gene, an F_2_ and a recombinant inbred line (RIL) population were derived from a cross between inbred line T877 (PSH) and DH1M (GSH). The *PSH* locus was mapped to the genomic region within 128.8 to 138.4 Mb using a bulked segregant sequencing approach. This position was further validated by linkage mapping using 190 F_2_ plants with GSH. Subsequently, the *PSH* locus was fine-mapped into an interval of 304.2 kb. A maize gene, GRMZM5G822829, was identified in this region, encoding a bHLH transcription factor. The expression level of this gene in T877 was found to be 9-fold higher than that of DH1M. In conclusion, our results suggest that GRMZM5G822829 is the putative candidate gene conferring leaf sheath color in maize.

## Introduction

The characteristic red, blue, and purple coloration in various plant tissues is due to anthocyanin accumulation [1]. Anthocyanin is a water-soluble natural pigment that belongs to the flavonoid class and occurs widely in plants. Anthocyanin plays important roles in plant growth and development, including the regulation of hormone responses [2], protection from damage by UV light [3], and defense against biotic and abiotic stresses [1]. Moreover, anthocyanin phenotypes, which are easily visually detected, have long been used in purity identification of hybrid seeds and as a useful reporter of transformation. A visible phenotypic marker, such as the purple leaf sheath, has many obvious advantages in conventional and transgenic breeding processes and is an important trait for maize breeding.

The biosynthesis of anthocyanin in maize is mainly focused on seed and plant tissues. Several structural genes (*c2*, *chi1*, *pr1*, *f3h*, *a1*, *a2*, *bz1* and *bz2*) interact with two types of transcription factors, the MYB-related protein (*c1*/*pl1*) and a bHLH-containing protein (*r1*/*b1*), to regulate patterns of pigmentation in maize tissues [4]. Although the general process of anthocyanin synthesis in maize, and particularly in seeds, is well understood, its regulation in the leaf sheath remains unclear. Several studies on the genetic control of leaf sheath color have been conducted in rice, the purple leaf sheath has been studied by linkage analysis in different segregation populations, and several leaf sheath genes have been fine-mapped and cloned. The leaf sheath color was controlled by one gene [5], while two main-effect QTLs involved in epistasis were identified for the purple leaf sheath in the Zhenshan97 × IRAT109 RIL population [6]. A single dominant gene governing purple leaf sheaths (PSH) was delimitated to 23.5 kb on chromosome 1 [7]. Another single dominant gene governing PSH was fine-mapped to a 153-kb interval on chromosome 6, and further sequencing and expression analyses revealed that *OsC1* was the candidate gene [8].

Gene/QTL mapping is a high-efficiency approach for the genetic dissection of qualitative and quantitative traits, which is the first step in map-based cloning of related genes and marker-assisted selection (MAS) in plant breeding. The traditional mapping strategy based on a bi-parental cross is labor-intensive, time-consuming, and costly [9]. Bulked-segregant analysis (BSA) is an elegant method to rapidly identify markers that are tightly linked to the causal gene(s) for a given phenotype [10,11]. However, the availability of DNA markers has limited the effectiveness of this method. With the rapid development of high-throughput genotyping based on next-generation sequencing (NGS), new approaches such as QTL-seq, combined with whole-genome re-sequencing with BSA, have been used to rapidly identify genes/QTLs in rice [12], oilseed rape [13], chickpea [14,15], and cucumber [16]. In this study, we conducted BSA and fine-mapping to narrow *PSH*, a dominant gene for purple leaf sheath from inbred line T877, to a 304.2 kb interval.

## Materials and methods

### Plant materials and phenotyping

To map the *PSH* locus, the inbred line T877, which has a purple leaf sheath, was crossed with the inbred line DH1M, which has a green leaf sheath. The F_1_ plants were self-crossed to develop the F_2_ mapping population, and a recombinant inbred line (RIL) population consisting of 208 F_8_ lines was developed using a single-seed descent method. A total of 190 recessive green leaf sheath (GSH) individuals from three ears were selected for the initial mapping, and a total of 831 plants with recessive phenotype (GSH) from the F_2_ population were used for fine mapping. For BSA, two parental pools and one GSH pool (lines with green leaf sheaths that were randomly selected from the RIL population) were constructed by mixing an equal amount of DNA from 30 individual samples. The mapping population and parental lines were grown together in a planting tray with 14 × 7 wells in the greenhouse of Yangzhou University (N32°23′, E119°25′) in the fall of 2014. The tray was filled with light nutritional soil. The leaf sheath color was identified at the third-leaf stage.

### Development of molecular markers and genotyping

Leaf tissue from each plant was harvested at the third-leaf stage, and genomic DNA was extracted by the CTAB method [17] with a minor modification. A total of 190 recessive green leaf sheath (GSH)-expressing individuals were genotyped using the MaizeSNP3K on the Illumina GoldenGate SNP genotyping platform at the National Maize Improvement Center of China at China Agricultural University. SNPs with poor quality were excluded from further analysis (S1 File). For PCR-based marker genotyping, PCR amplification products were analyzed on 1% agarose gels or 6% denaturing polyacrylamide gels.

In this study, PCR markers were retrieved from the Maize Genetics and Genomics Database (http://www.maizegdb.org/; S1 Table). New indel (insertion or deletion) markers were developed based on the sequence difference between DH1M and T877 using the resequencing data of the parents. Primers for the indel markers were designed with Primer 5 (S2 Table).

### NGS-based whole-genome sequencing and BSA analysis

A total of three Illumina libraries (two parental pools and one GSH pool) were constructed. Pair-end sequencing libraries (read length 100 bp) with insert sizes of approximately 500 bp were prepared for sequencing with an Illumina HiSeq 2000 machine. The genomic DNA was quantified on a Qubit 2.0 and set to an equal concentration. A size selection of the libraries was performed using a 2% agarose gel to obtain a target insert size of 500–600 bp and purified for further analysis. The size distribution of the amplified DNA libraries was confirmed using an Agilent Technologies 2100 Bioanalyzer with a high-sensitivity chip. The libraries were enriched using adapter-compatible PCR primers.

The quality of the raw sequence reads was confirmed using FASTQC v0.10.1, and the adapters and low-quality reads were filtered. The filtered high-quality sequences were aligned and mapped onto the B73 reference genome (ftp://ftp.ensemblgenomes.org/pub/plants/release-24/fasta/zea_mays/dna/) using BWA software [18]. SNP calling was performed using SAMtools software [18]. Low-quality SNPs with a base quality value <20 and a read depth <4× the coverage from the GSH pool sequences were excluded. SNP-index values [12,19] were calculated to identify candidate regions for the purple leaf sheath gene. A SNP-index is the proportion of reads harboring the SNP that are different from the purple parent (T877) sequence. The SNP-index for each SNP position was calculated for the GSH-pool using the following formula: SNP-index (at a position) = (count of reads aligned with the green sheath parent DH1M) / (count of reads aligned in the GSH-pool). The average SNP-index of the SNPs located in each genomic interval was calculated using a sliding window, with a 1 Mb window size with 10 kb increments. The SNP-index graphs for the GSH-pool were plotted.

### Trait-marker association analysis in the F_2_ population

The F_2_ population included 190 plants that were recessive for the green leaf sheath trait. The segregation ratio of markers that are unlinked with the phenotype should be 1:2:1 (T877 homozygotes: heterozygotes: DH1M homozygotes). If the marker is in the particular genomic region harboring the candidate gene, the segregation ratio would show partial segregation to the DH1M (GSH inbred line) allele. The SNP-trait relationship was tested for 1369 SNPs with polymorphisms distributed across the genome using Fisher’s exact test. The significance threshold was corrected for multiple comparisons using the Bonferroni correction, α′≈α/n = 0.05/1369 = 3.6 × 10^−5^, where α is the nominal significance threshold and *n* is the number of SNPs. To eliminate the influence of partial separation markers that were associated with the phenotype, a sliding window with 5 SNPs was applied to calculate the average value and standard deviation of the *χ*^2^, and the abnormal SNPs were eliminated.

### RNA isolation, reverse transcription, and qRT-PCR analysis

The leaves, sheaths, stems, and roots at three leaf stages with three replicates from both the DH1M and T877 lines were collected for qRT-PCR analysis. Total RNA was isolated from different tissues using an RNA Isolation Kit (Tiangen Biotech). High-quality first-strand cDNA was generated using oligo (dT) and PrimeScript^™^ II (Takara). Gene-specific primers (PSH-F: 5’-ACTGCTTCCGTCCATTCAC, PSH-R: 5’-ACAGACCTCCTTCCTCACAC) were designed according to the gene sequence using QuantPrime (http://www.quantprime.de/). The relative expression levels of the gene were calculated using the 2^−ΔΔCt^ method [20,21], which represents the CT (cycle threshold) difference between the reference Actin gene (GRMZM2G126010) and the target gene product.

## Results

### The purple leaf sheath trait is controlled by a dominant gene

**Fig 1:**
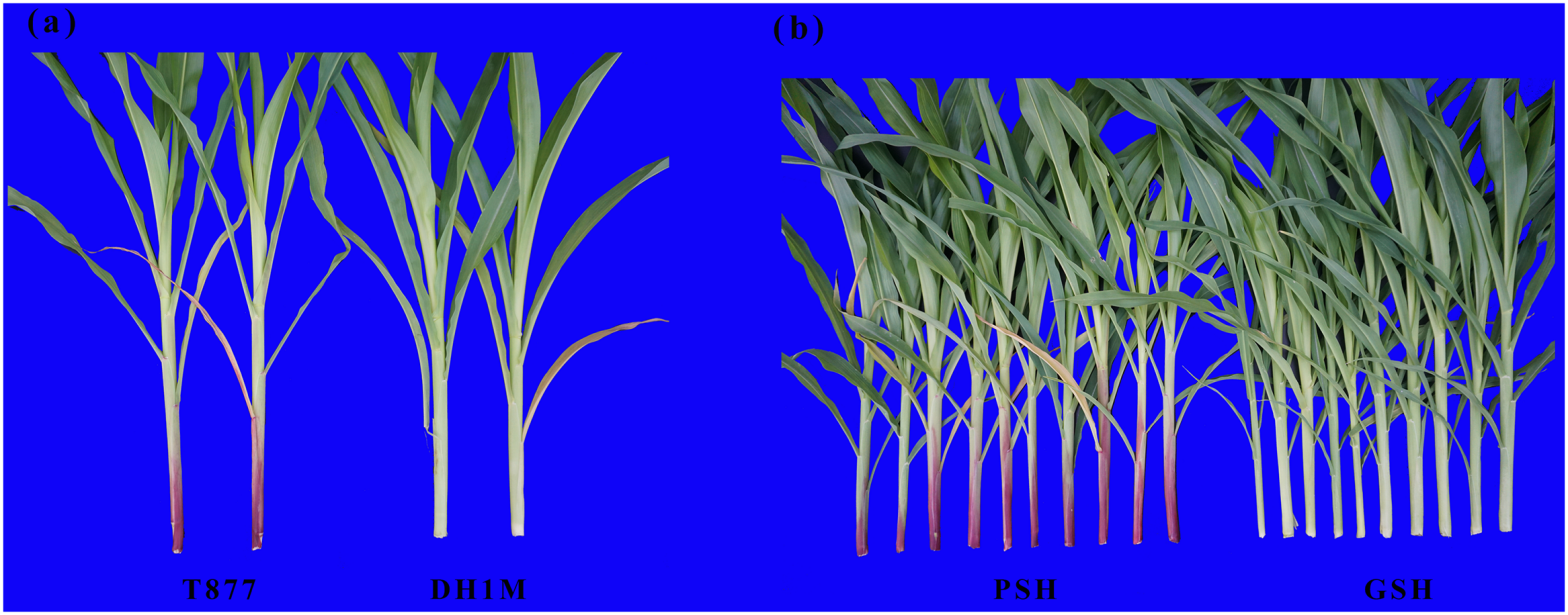
Phenotypes of parental inbred lines (a) and progeny lines in RIL population (b) in sixth leaf stage.

The inbred line T877 showed purple leaf sheaths, coleoptile, and apiculus; the roots that were exposed to light also remained purple. However, the DH1M inbred line displayed green leaf sheaths (Fig 1a; Panel a and b in S1 Fig). Both the T877 and DH1M lines had green leaf blades for their entire life cycle (Panel a, b, c and d in S1 Fig). In the F_2_ population, there was a total of 3,257 progenies, including 2,426 plants with PSH and 831 plants with GSH. The segregation ratio was 2.92:1, which fit the Mendelian segregation ratio of 3:1 (*χ*^2^ = 0.67 < *χ*^2^_0.05,1_ = 3.84). This indicates that the PSH trait is controlled by a major gene and that PSH is dominant over GSH. In addition, 83 plants with PSH and 86 GSH lines were identified in the RIL population. The ratio of PSH to GSH also followed single-gene Mendelian inheritance, confirming the result observed in the F_2_ population. Ten RILs with purple leaf sheaths and 10 RILs with green leaf sheaths were randomly selected and are shown in Fig 1b. The two bulks exhibit an obvious difference in leaf sheath color.

### Identification and mapping of the gene for purple leaf sheath using the bulked segregant sequencing approach

**Fig 2:**
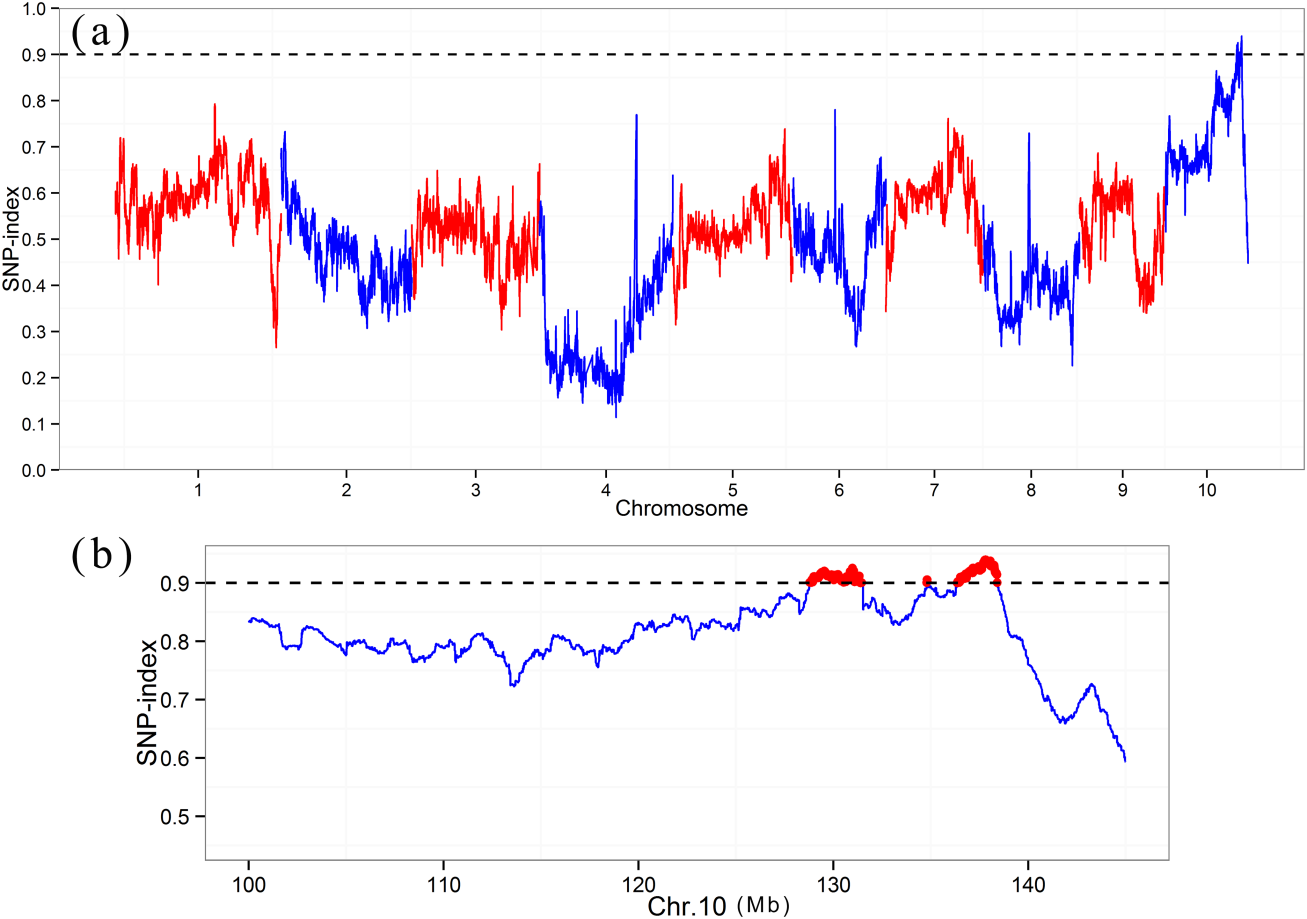
Single nucleotide polymorphism (SNP)-index plots across the whole genome (a) and chromosome 10 (b).

To map the *PSH* locus, three Illumina libraries (two parents and a green GSH pool) were constructed and sequenced using the Illumina HiSeq 2000. High-throughput sequencing resulted in 231,000,883, 247,471,260, and 715,731,102 short reads (100 bp in length) from the T877 pool (11.9× depth coverage), DH1M pool (12.2× depth coverage), and GSH pool (33.2× depth coverage), respectively (Table 1). After filtering out adapter and low-quality reads, the clean reads were aligned to the B73 reference genome and 489,668 high-quality SNPs were identified between the two parents. Using the GSH parent DH1M as a reference, the SNP-index of the GSH pool was calculated across the genome. Accordingly, a SNP-index of more than 0.9 indicates a significant contribution of that SNP to the *PSH* locus. The average SNP-index was estimated in a 1-Mb genomic interval using a 10-kb sliding window. A SNP-index graph was generated by plotting the average SNP-index against the position in the maize genome (Fig 2a). Two major genomic regions and one locus on chromosome 10 (128.8–131.5 Mb, 134.8 Mb and 136.4–138.4 Mb) had a SNP-index higher than 0.9, and the region from 136.4 to 138.4 Mb had the highest SNP-index (Fig 2b). A detailed analysis of this target genomic region indicated that the GSH individuals contained most of the SNP alleles from the DH1M line (S3 Table).

**Table 1:** Statistics of sequencing depth and coverage.

Sample	Raw reads	Clean reads	Mapped reads	Mapping ratio(%)	Coverage (×)
T877	231,000,883	228,443,925	180,027,057	98.51	11.85
DH1M	247,471,260	244,649,833	192,805,437	98.51	12.21
GSP	715,731,102	706,840,180	557,315,361	98.56	33.21

### Validation of gene mapping through the traditional linkage method

**Fig 3:**
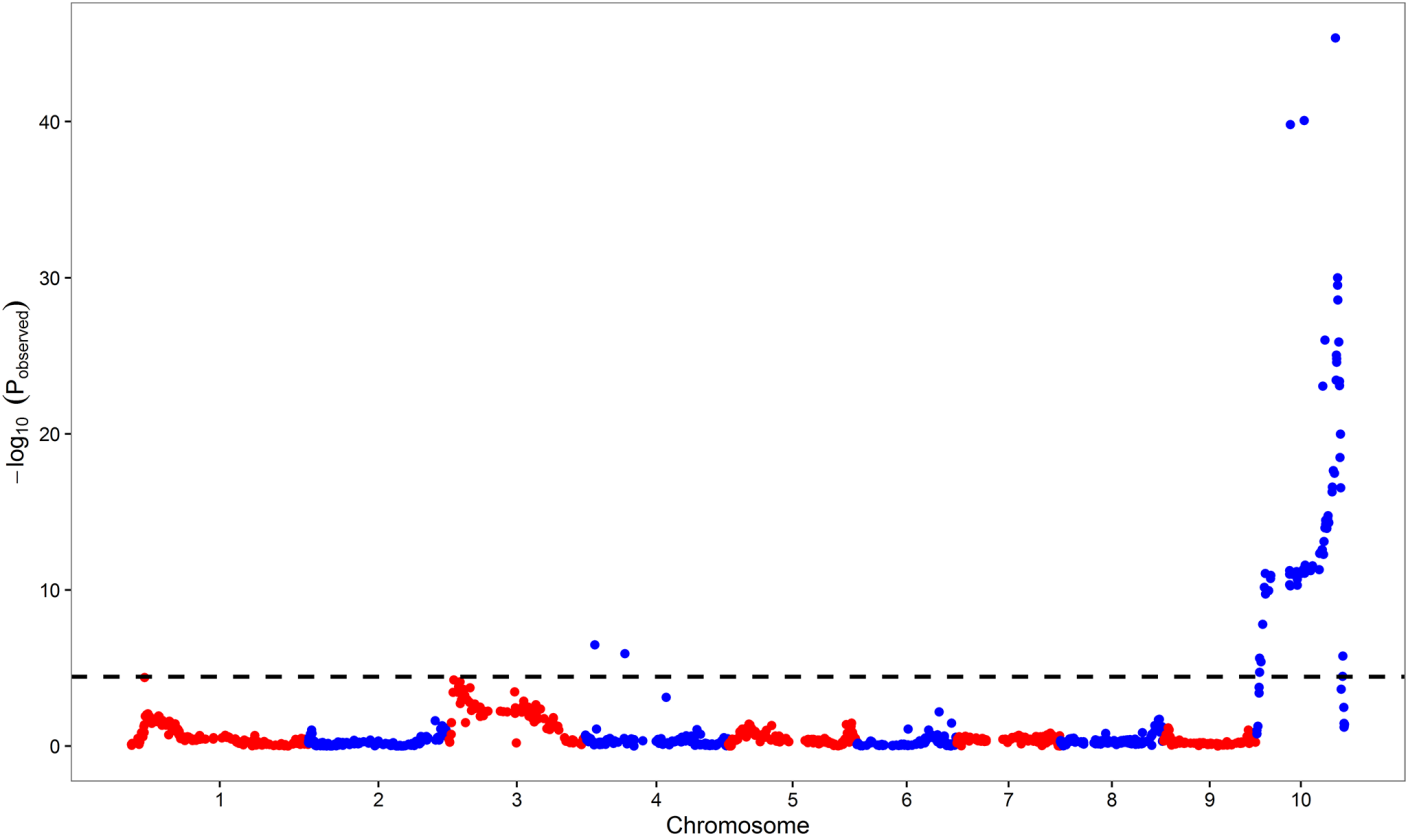
DNA chip screening across 190 F_2_ plants mapped the *PSH* locus onto chromosome 10. The black dashed line represents the 5% significance threshold with a Bonferroni correction.

To determine the accuracy of the bulked segregant sequencing mapping result, traditional gene mapping was also performed. A total of 190 F_2_ individuals with GSH and the parental lines were genotyped with the MaizeSNP3K BeadChip. After removing poor-quality SNPs, 1,369 SNPs were found to be polymorphic between the parents. A total of 68 SNPs showed a significant association with PSH traits using Fisher’s exact test, including 66 and 2 SNPs on chromosomes 10 and 4, respectively (Fig 3). The most significant SNP was SYN17666 (-log_10_*P* = 45.4; Table 2) on chromosome 10. The SNPs around SYN17666 were all significantly associated with PSH traits and the candidate region spanned 7 Mb (chr10: 134,411,828–141,375,429; Fig 3; Table 2). SYN17666 was converted to PCR-based marker CAPS01 for screening recombinants. This result was consistent with that obtained from bulked segregant sequencing analysis, supporting the finding that a gene for purple leaf sheath is located approximately in the 128.8–141.4 Mb genome interval of chromosome 10.

**Table 2:** SNPs significantly associated with purple leaf sheath (PSH).

SNP	Chr.	Position^a^	Homo^b^	Hete^c^	Homo^d^	*P* value^e^	-log_10_*P*	Annotation
PZE-110026107	10	58,673,508	175	1	14	1.53E-40	39.81	intergenic
SYN9732	10	82,025,778	173	1	11	8.54E-41	40.07	GRMZM5G832300
SYN17666	10	134,411,828	184	1	4	4.33E-46	45.36	GRMZM2G016939
PZE-110082278	10	135,577,726	146	39	5	3.62E-24	23.44	intergenic
PZE-110083952	10	136,247,247	150	35	5	9.21E-26	25.04	GRMZM2G305146
PZE-110084185	10	136,444,745	149	35	5	1.55E-25	24.81	intergenic
PZE-110084754	10	136,713,611	149	34	6	2.63E-25	24.58	intergenic
PZE-110087471	10	138,051,369	159	28	3	3.07E-30	29.51	GRMZM2G360529
PZE-110087783	10	138,222,747	159	26	3	1.00E-30	30.00	intergenic
PZE-110088338	10	138,665,174	155	28	3	2.69E-29	28.57	intergenic
SYN37373	10	139,995,358	152	33	5	1.32E-26	25.88	GRMZM2G128092
PZE-110093304	10	141,297,647	146	37	6	4.41E-24	23.36	GRMZM2G391042
SYN38150	10	141,375,429	144	36	6	8.14E-24	23.09	GRMZM2G128934

^a^Position in base pairs for the lead SNP according to version 5b.60 of the maize reference.

^b^Homozygous for DH1M (green leaf sheath).

^c^Heterozygous for both alleles.

^d^Homozygous for T877 (purple leaf sheath).

^e^*P* value of Fisher’s exact test.

### Fine mapping of *PSH* locus

**Fig 4:**
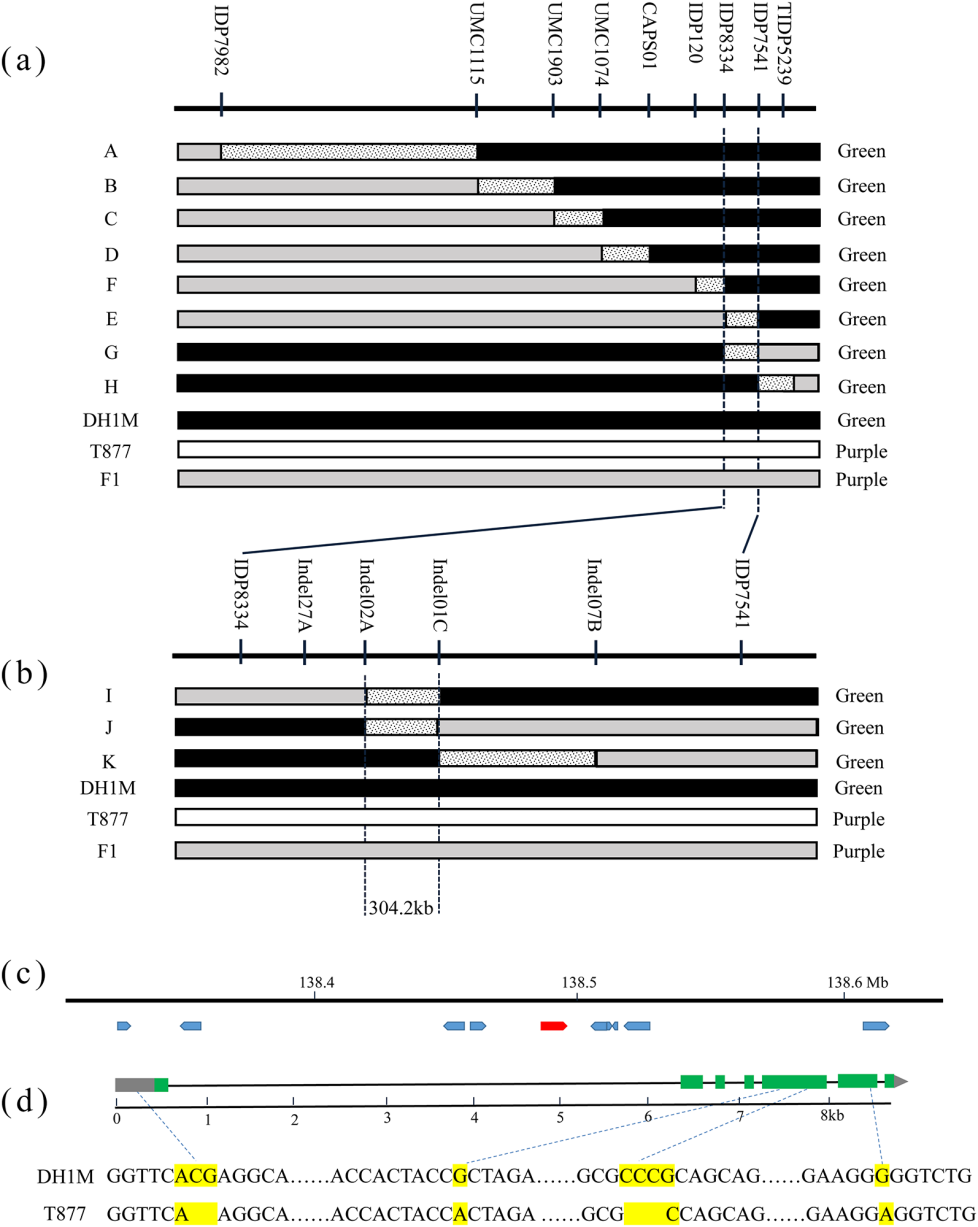
Isolation and sequence analysis of the gene conferring responsible for purple leaf sheath. (a) Fine mapping the *PSH* locus to the region between markers IDP7541 and TIDP5239. (b) Fine mapping narrows down the *PSH* locus to 304.2 kb between markers Indel02A and Indel01C. (c) The predicted genes in the 304.2 kb genomic region. (d) Gene structure of *PSH* and sequence analysis of T877 and DH1M.

To fine-map the *PSH* gene, eight polymorphic PCR markers around SYN17666 were identified from the MaizeGDB database (S1 Table). A total of 831 plants with the recessive phenotype (GSH) from the F_2_ population of 3,257 individuals were selected for recombinant screening. All selected plants were genotyped using IDP7982 and TIDP5239, which identified a total of 39 recombinants on both sides. Using these polymorphic markers to screen these recombinants, the 39 recombinants were grouped into 8 groups; all of the recombinants showed green leaf sheath, recessive phenotype (Fig 4a). In group A, the crossover point appeared between IDP7982 and UMC1115; recombinant individuals were found to have the heterozygous allele with IDP7982 and the homozygous susceptible allele between UMC1115 and TIDP5239. Group A placed the gene for purple leaf sheath in a region downstream of IDP7982. The reciprocal group H, which contained recombinant individuals between IDP7541 and TIDP5239, placed the gene in a region upstream of TIDP5239. Using the same procedure, the genomic region containing the gene was further narrowed to an interval bounded by IDP8334 and IDP7541.

To more precisely determine the critical recombination point, four polymorphic indel markers (Indel01C, Indel02A, Indel07B and Indel27A; S2 Table) were developed based on resequencing data from the parental genomes. Using these polymorphic markers, three new group recombinants were identified (Fig 4b). Groups I and J contained recombinant individuals between Indel02A and Indel01C and group K contained recombinant individuals between Indel01C and Indel07B. These results demonstrated that the *PSH* locus must reside in the recombination region between markers Indel02A and Indel01C. Thus, the *PSH* locus was narrowed down to a region flanked by the markers Indel02A and Indel01C; this region had a physical length of 304.2 kb according to the B73 reference sequence

### Candidate genes prediction

Gene prediction analysis of the 304.2 kb DNA fragment using the B73 genome sequence identified 10 putative open reading frames (ORFs; Table 3; Fig 4c). Sequence annotation analysis indicated that ORF5 (GRMZM5G822829), which consists of 7 exons encoding a bHLH transcription factor, was the most likely gene (Table 3; Fig 4c). Sequence comparison of ORF5 between DH1M and T877 revealed four polymorphisms (Fig 4d), including 2 indels and 2 SNPs. In INDEL1, two nucleotides are missing in a transition from ACG (DH1M) to A (T877) in the promoter region. INDEL2 transformed CCCG to C in the fifth exon, leading to a loss of alanine. SNP1, a single nucleotide transition from G (DH1M) to A (T877), is present in the fifth exon, and SNP2, a single nucleotide transition from G (DH1M) to A (T877), is present in the sixth exon (Fig 4d). SNP1 does not cause an amino acid residue change; however, the transition in SNP2 causes an amino acid residue change from glycine to glutamate. SNP1, Indel1 and Indel2 in the candidate gene were tested in the RIL population and found that these markers co-segregated with the trait (S4 Table).

**Table 3:** Candidate genes in *PSH* region.

Candidate gene	Location	Arabidopsis best hit	Annotations
GRMZM2G105801	Chr10:138324276–138326264	AT4G00525	Unknown
GRMZM2G344476	Chr10:138344377–138349103	AT1G01710	Acyl-CoA thioesterase family protein
AF466202.2_FG001	Chr10:138456052–138460451	AT2G19900	(ATNADP-ME1, NADP-ME1) NADP-malic enzyme 1
GRMZM5G865471	Chr10:138463098–138465695	AT5G43810	(AGO10, PNH, ZLL) Stabilizer of iron transporter SufD / Polynucleotidyl transferase
GRMZM5G822829	Chr10:138489998–138498818	AT1G63650	(ATMYC-2, EGL1, EGL3) Basic helix-loop-helix (bHLH) DNA-binding superfamily protein
GRMZM5G803874	Chr10:138512812–138515509	AT4G11570	Haloacid dehalogenase-like hydrolase (HAD) superfamily protein
GRMZM5G838732	Chr10:138515896–138516198	None	None
AF466202.2_FG006	Chr10:138517754–138518621	AT1G11330	S-locus lectin protein kinase family protein
AF466202.2_FG007	Chr10:138520769–138527883	AT5G01670	NAD(P)-linked oxidoreductase superfamily protein
AF466202.2_FG008	Chr10:138620902–138629501	AT4G37860	SPT2 chromatin protein

**Fig 5:**
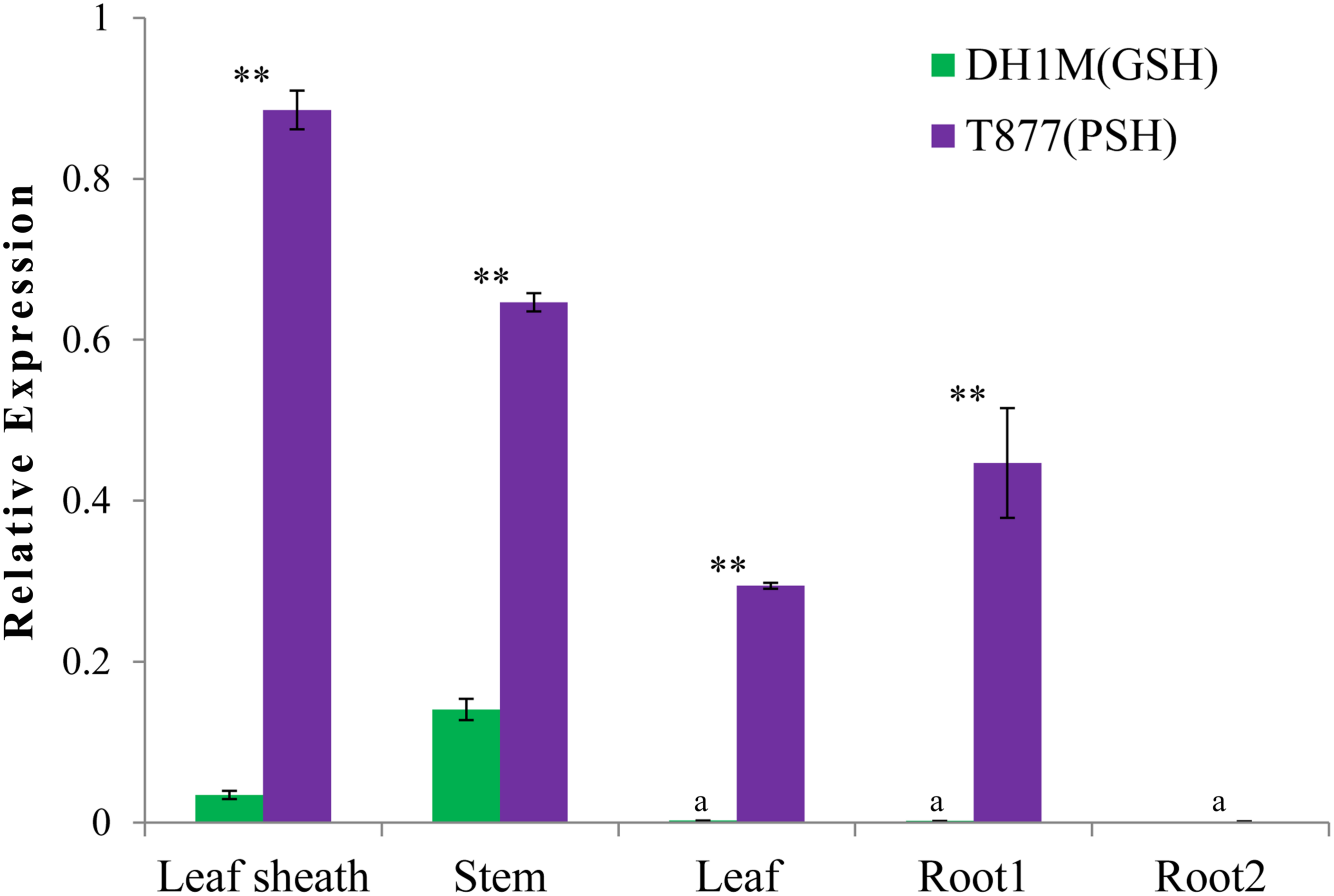
The relative expression levels of *PSH* in different tissues for T877 and DH1M. Root1 and root2 indicated roots exposed to light and in the dark, respectively. The lowercase letters “a” indicate that the gene is almost non-expressed, “**” indicate that a significant difference was detected between T877 and DH1M in the same tissue sample (*p*<0.01).

Real-time PCR was performed to determine the expression pattern of ORF5. RNA was isolated from the leaf sheath, stem, leaf, light-exposed roots and dark-exposed roots of two parents at the third-leaf stage. The expression level of GRMZM5G822829 was significantly higher in line T877 than in line DH1M in the leaf sheath, stem, leaf, and roots exposed to light. The expression level was highest in the leaf sheath, and a nearly 9-fold expression difference was observed between T877 and DH1M (Fig 5). GRMZM5G822829 showed almost no expression in dark-exposed roots, indicating that the expression of GRMZM5G822829 can be induced by light. The expression pattern of GRMZM5G822829 was consistent with the phenotype difference between the two parents. These findings indicate that GRMZM5G822829 is a potential candidate gene regulating the purple leaf sheath trait in maize, although a further genetic transformation assay needs to be performed for functional validation of the gene.

## Discussion

Genetic mapping of QTLs/genes associated with economically important traits is important for the efficient development of new and elite lines in plant breeding programs. However, traditional QTL mapping, which requires the development of polymorphic markers between parental lines, is time-consuming and labor-intensive [22]. New sequencing technologies and high throughput platforms combine the advantages of time- and cost-effectiveness and dense marker coverage and open opportunities to redesign gene mapping strategies [23]. A bin-map strategy based on whole-genome resequencing or genotyping by sequencing (GBS) could greatly improve mapping accuracy and resolution [23,24]. The NGS-based QTL-seq approach, which is a cost-effective and rapid method, can rapidly identify small genomic regions associated with the target trait without the tedious genotyping of a large population [12]. This method has become the most effective approach for quickly identifying the major QTLs controlling qualitative and quantitative traits in crop plants [12–16]. In this study, the leaf sheath color in maize was found to be controlled by a dominant gene, to reduce the scale and cost, two parental bulks and one GSH bulk were sequenced; the most significant region identified by the BSA method was approximately 240 kb from the target gene (Fig 2b). The BSA pools always contain a few individuals, so the obtained recombinants in the target region limit the accuracy of the gene mapping. It is necessary to conduct fine mapping to obtain the candidate gene. BSA also provided the polymorphism for designing PCR markers in the target region to complement fine mapping and cloning experiments. Here, four newly developed indel markers were used to screen new recombinants, and the gene was narrowed to a 304.2 kb genomic region (Fig 4) containing 10 genes. The combination of bulked segregant sequencing and traditional linkage analyses was a powerful approach for the rapid identification of a candidate gene in maize.

Anthocyanin accumulation in different tissues requires the action of many genes, including structural genes that encode biosynthetic enzymes and regulatory genes that regulate the patterns of anthocyanin pigmentation [4]. The structural genes are coordinately regulated by at least two groups of regulatory genes to influence the intensity and pattern of anthocyanin biosynthesis, which are responsible for the tissue-specific pigmentation of plants and seeds [25]. The *c1*/*pl1* family, encodes a MYB transcription factor. The *c1* gene induces pigmentation only in seed tissues, such as the aleurone and embryo [26], whereas *pl1* controls pigmentation in plant tissues, such as the plant body and pericarp. The second group is the *r1*/*b1* gene family, which encodes a basic helix-loop-helix (bHLH)-type transcription factor [27]. The *b1* alleles consist of a single gene, but the *r1* gene has many phenotypically diverse alleles and encodes a distinct pigmentation pattern [25,28]. The *r1* alleles activate anthocyanin (red and purple pigments) expression in a wide array of tissues, including the aleurone of the kernel, scutellum, pericarp, roots, husks, silks, leaves, anthers, coleoptile, leaf basis (ligule), and glumes [25,28–30]. Basic helix-loop-helix proteins (bHLHs) are one of the largest families of transcription factors in plants, and more than 630 bHLH genes have been identified [31]. The bHLH transcription factors are involved in many physiological processes, such as photomorphogenesis [32], hormone responses [33], and regulation of floral organ development [33]. The bHLH transcription factor involved in anthocyanin synthesis is a research topic of great interest. bHLH transcription factors are usually expressed together with MYB proteins to control the synthesis of anthocyanin [34]. In maize, a member of the b-HLH family (*b1* or *r1*) and a member of the MYB family (*c1* or *pl1*) are both required for anthocyanin pigmentation [35]. In apples, *MdMYB10* is dependent on the co-expression of two distinct bHLH proteins, MdbHLH3 and MdbHLH33, to control anthocyanin biosynthesis [36]. In *Arabidopsis*, *TT8* and *TT2* were found to code for a basic helix-loop-helix domain transcription factor and an R2R3 MYB domain protein, respectively, which are necessary for proanthocyanidin and anthocyanin biosynthesis in young seedlings [37]. Most of the regulatory genes in anthocyanin biosynthesis showed variable expression, such as *TT8* gene, which encodes a bHLH domain protein and is expressed throughout seed development and in young seedlings [38]. In this study, the *PSH* gene was expressed in leaf sheath, stem, leaf, and root that was exposed to light in T877; the variable expression in these tissues was basically consistent with different degrees of purple color.

External environmental factors, such as light, temperature, water availability, nutritional effects, pH, sugar and plant hormones, can regulate the biosynthesis and accumulation of anthocyanin in plants [39, 40]. Light is the most important of these factors. Strong light can induce the expression of anthocyanin-synthesis genes to increase anthocyanin accumulation. In maize, the expression of the *pl-bol3* gene (encoding an MYB-related transcription factor) is induced by light and the expression of the bHLH (basic helix-loop-helix) *Sn1-bol3* gene is stimulated by several light qualities [41]. Here, the gene expression of the root that was exposed to light was significantly higher than that of the root in the dark, indicating that the *PSH* gene was strongly induced by light (Fig 5).

Here, we identified the potential candidate gene for purple leaf sheath, a trait that has been widely used as a phenotypic marker in maize. For this trait, even phenotypic screening is high-efficiency, when whole-genome scans are being used, purple leaf sheath can be selected for by molecular markers if the corresponding gene has been identified, and the selection could be carried out before sowing to eliminate undesirable plant genotypes [42]. In summary, in the current study, we combined a BSA and traditional QTL mapping strategy to fine-map the gene for the purple leaf sheath trait. The PSH locus was delimited within a 304.2 kb region in the maize genome harboring ten candidate genes. A sequence comparison and gene expression analysis indicated that GRMZM5G822829 is the putative candidate gene for the purple leaf sheath trait.

## Supporting information

S1 Table The list of 8 PCR markers screened from MaizeGDB. (DOCX)

S2 Table The list of 4 indel markers newly developed based on resequencing data of the parents. (DOCX)

S3 Table Candidate SNPs for purple leaf sheath identified by QTL-seq. (DOCX)

S4 Table Recombinants screening in the RIL population. (DOCX)

S1 Fig Phenotypes of parental inbred lines in different stages. (a) three-leaf stage, (b) six-leaf stage, (c) T877 in adult stage, (d) DH1M in adult stage.
(PDF)

S1 File Genotype for F_2_ population. (CSV)
