# Correction: Combined bulked segregant sequencing and traditional linkage analysis for identification of candidate gene for purple leaf sheath in maize

**DOI:** 10.1371/journal.pone.0196296

**Published:** 2018-04-18

**Authors:** Pengcheng Li, Cancan Du, Yingying Zhang, Shuangyi Yin, Enying Zhang, Huimin Fang, Dezhou Lin, Chenwu Xu, Zefeng Yang

The following information is missing from the Funding section: This work was supported by grants from National Key Technology Research and Development Program of MOST-2016YFD0100300, The National Natural Science Foundations- 31601810, The Priority Academic Program Development of Jiangsu Higher Education Institutions, The Natural Science Foundations of Jiangsu Province-BK20150010, Natural Science Foundation of the Jiangsu Higher Education Institutions-14KJA210005, Innovative Research Team of Universities in Jiangsu Province.
