# Supplementary material for: Combined bulked segregant sequencing and traditional linkage analysis for identification of candidate gene for purple leaf sheath in maize
Source: PLoS One. 2018 Jan 5;13(1):e0190670. doi: 10.1371/journal.pone.0190670 (PMC5755806; doi:10.1371/journal.pone.0190670)
Supplement: S1 Table — (DOCX) [file pone.0190670.s001.docx]

S1 Table. The list of 8 PCR markers screened from MaizeGDB

| Marker | Name | Position | Sequence |
| --- | --- | --- | --- |
| IDP7982 | IDP7982-F | 108,118,305 | TGATTTCACCCATGTGTTGC |
|  | IDP7982-R |  | ACGACGGCTTTCAGATATGG |
| umc1115 | umc1115-F | 126,624,613 | TGGAAGGGGATATCAGGATTTAGA |
|  | umc1115-R |  | TGTGATGACCATGAATGTAAGCTG |
| umc1930 | umc1930-R | 130,287,456 | CACACAGTGAGTCGTTTCTTTCGT |
|  | umc1930-F |  | TCTTCTCCAAGTGTGTTAATGCCC |
| umc1074 | bmc1074-R | 130,965,990 | TTTCCCCCTGATTCGTTATG |
|  | bmc1074-F |  | CATGCTAATAGCCTACCGGG |
| IDP120 | IDP120-F | 136,877,687 | TTCGTCAGCAGTTAAGCACG |
|  | IDP120-R |  | GTTCACCAACAAGGCGTACC |
| IDP8334 | IDP8334-F | 137,827,877 | CTCAACCAAGAGAGCGTGC |
|  | IDP8334-R |  | GAGATGATTCTCCAGGCTGC |
| IDP7541 | IDP7541-F | 139,859,989 | AACGTGTACGACCTTACGCC |
|  | IDP7541-R |  | TACCAAGTACCAACCCTCGC |
| IDP5239 | TIDP5239-F | 142,068,660 | TTGGTTTCAAGACTCCCAGG |
|  | TIDP5239-R |  | TTCTGCCTCTGATAGAAACGG |
