# Supplementary material for: Combined bulked segregant sequencing and traditional linkage analysis for identification of candidate gene for purple leaf sheath in maize
Source: PLoS One. 2018 Jan 5;13(1):e0190670. doi: 10.1371/journal.pone.0190670 (PMC5755806; doi:10.1371/journal.pone.0190670)
Supplement: S2 Table — (DOCX) [file pone.0190670.s002.docx]

S2 Table. The list of 4 InDel markers newly developed based on resequencing data of parents

| Marker | Name | Position | Sequence |
| --- | --- | --- | --- |
| Indel27A | Indel27A-F | 138,124,023 | AAATGAACAGAATCAGACGG |
|  | Indel27A-R |  | TTGTGGTGGAAGTGAGAGA |
| Indel02A | indel02A-F | 138,325,278 | GAAACCCACAAGAAACAAGG |
|  | indel02A-R |  | CAACAGACCAATACAAGCGA |
| Indel01C | indel01C-F | 138,629,440 | TTGAGGAAGAAGAGAGACG |
|  | indel01C-R |  | GTGGTAAGGGTAAACAGGA |
| Indel07B | indel07B-F | 139,264,294 | ACCGCCAGACCACTGACAT |
|  | indel07B-R |  | AATCCTTCGCCACAGCCTC |
