# Supplementary material for: Combined bulked segregant sequencing and traditional linkage analysis for identification of candidate gene for purple leaf sheath in maize
Source: PLoS One. 2018 Jan 5;13(1):e0190670. doi: 10.1371/journal.pone.0190670 (PMC5755806; doi:10.1371/journal.pone.0190670)
Supplement: S3 Table — (DOCX) [file pone.0190670.s003.docx]

S3 Table. Candidate SNPs for purple leaf sheath identified by BSA analysis

| CHR | POS | Gen  _T877^1^ | Gen  _DH1M^2^ | T877  _depth  _GSP^3^ | DH1M  _depth  _GSP^4^ | Depth  _GSP^5^ | SNP-index |
| --- | --- | --- | --- | --- | --- | --- | --- |
| 10 | 136502620 | C | T | 0 | 26 | 26 | 1 |
| 10 | 136644089 | C | T | 0 | 19 | 19 | 1 |
| 10 | 136675593 | C | T | 0 | 10 | 10 | 1 |
| 10 | 136727627 | T | C | 0 | 14 | 14 | 1 |
| 10 | 136781987 | T | A | 0 | 12 | 12 | 1 |
| 10 | 136790458 | A | C | 0 | 14 | 14 | 1 |
| 10 | 136801426 | C | T | 0 | 29 | 29 | 1 |
| 10 | 136801427 | A | C | 0 | 29 | 29 | 1 |
| 10 | 136801444 | C | T | 0 | 27 | 27 | 1 |
| 10 | 136801473 | G | A | 0 | 34 | 34 | 1 |
| 10 | 136801484 | A | T | 0 | 35 | 35 | 1 |
| 10 | 136801487 | G | T | 0 | 35 | 35 | 1 |
| 10 | 136827778 | A | G | 0 | 7 | 7 | 1 |
| 10 | 136830147 | T | C | 0 | 17 | 17 | 1 |
| 10 | 136831677 | T | C | 0 | 60 | 60 | 1 |
| 10 | 137190588 | C | A | 0 | 21 | 21 | 1 |
| 10 | 137191585 | C | T | 0 | 26 | 26 | 1 |
| 10 | 137191590 | T | A | 0 | 26 | 26 | 1 |
| 10 | 137357197 | G | T | 0 | 26 | 26 | 1 |
| 10 | 137379081 | G | A | 0 | 29 | 29 | 1 |
| 10 | 137379087 | C | A | 0 | 30 | 30 | 1 |
| 10 | 137486483 | G | A | 0 | 10 | 10 | 1 |
| 10 | 137486484 | T | C | 0 | 8 | 8 | 1 |
| 10 | 137486496 | T | C | 0 | 12 | 12 | 1 |
| 10 | 137524934 | A | G | 0 | 20 | 20 | 1 |
| 10 | 137525371 | A | G | 0 | 25 | 25 | 1 |
| 10 | 137525378 | G | A | 0 | 25 | 25 | 1 |
| 10 | 137525381 | C | A | 0 | 26 | 26 | 1 |
| 10 | 137525417 | T | C | 0 | 36 | 36 | 1 |
| 10 | 137563683 | C | T | 0 | 19 | 19 | 1 |
| 10 | 137683755 | C | T | 0 | 20 | 20 | 1 |
| 10 | 137685692 | C | T | 0 | 18 | 18 | 1 |
| 10 | 137685704 | C | T | 0 | 17 | 17 | 1 |
| 10 | 137685876 | T | C | 0 | 21 | 21 | 1 |
| 10 | 137685878 | A | G | 0 | 21 | 21 | 1 |
| 10 | 137780533 | G | A | 0 | 41 | 41 | 1 |
| 10 | 137780741 | G | A | 0 | 44 | 44 | 1 |
| 10 | 137784854 | C | T | 0 | 26 | 26 | 1 |
| 10 | 137843115 | A | T | 0 | 16 | 16 | 1 |
| 10 | 137852778 | G | C | 0 | 25 | 25 | 1 |
| 10 | 137908311 | C | T | 0 | 16 | 16 | 1 |
| 10 | 137908362 | A | T | 0 | 14 | 14 | 1 |
| 10 | 137926854 | A | G | 0 | 16 | 16 | 1 |
| 10 | 137979180 | T | A | 0 | 27 | 27 | 1 |
| 10 | 138070382 | G | A | 0 | 23 | 23 | 1 |
| 10 | 138071035 | C | G | 0 | 47 | 47 | 1 |
| 10 | 138216934 | G | A | 0 | 14 | 14 | 1 |
| 10 | 138240242 | A | C | 0 | 12 | 12 | 1 |
| 10 | 138240255 | A | C | 0 | 13 | 13 | 1 |
| 10 | 138240259 | T | C | 0 | 14 | 14 | 1 |
| 10 | 138243857 | T | C | 0 | 13 | 13 | 1 |
| 10 | 138244299 | A | C | 0 | 8 | 8 | 1 |
| 10 | 138244300 | T | G | 0 | 8 | 8 | 1 |
| 10 | 138327258 | T | C | 0 | 33 | 33 | 1 |
| 10 | 138335640 | T | G | 0 | 13 | 13 | 1 |
| 10 | 138335648 | G | A | 0 | 13 | 13 | 1 |
| 10 | 138450972 | A | G | 0 | 19 | 19 | 1 |
| 10 | 138478431 | G | A | 0 | 8 | 8 | 1 |
| 10 | 138481507 | C | T | 0 | 23 | 23 | 1 |
| 10 | 138498966 | A | G | 0 | 16 | 16 | 1 |
| 10 | 138498970 | A | G | 0 | 16 | 16 | 1 |
| 10 | 138530809 | C | A | 0 | 25 | 25 | 1 |
| 10 | 138530840 | T | G | 0 | 25 | 25 | 1 |
| 10 | 138534090 | G | A | 0 | 17 | 17 | 1 |
| 10 | 138622107 | A | G | 0 | 23 | 23 | 1 |

1:Genotype of T877

2:Genotype of DH1M

3:The number of reads that is the same as T877 in GSP

4: The number of reads that is the same as DH1M in GSP

5: The number of reads in this locus in GSP
