# Supplementary material for: Combined bulked segregant sequencing and traditional linkage analysis for identification of candidate gene for purple leaf sheath in maize
Source: PLoS One. 2018 Jan 5;13(1):e0190670. doi: 10.1371/journal.pone.0190670 (PMC5755806; doi:10.1371/journal.pone.0190670)
Supplement: S4 Table — (DOCX) [file pone.0190670.s004.docx]

S4 Table. Recombinants screening in the RIL population

| Recombinants | Marker | | | | | | | | | | | | | | | Phenotype |
| --- | --- | --- | --- | --- | --- | --- | --- | --- | --- | --- | --- | --- | --- | --- | --- | --- |
|  | UMC  1115 | UMC  1930 | UMC  1074 | CAPS01 | IDP  120 | IDP  8334 | Indel27A | Indel02A | Indel1 | Indel2 | SNP2 | Indel01C | Indel07B | IDP  7541 | TIDP5239 |  |
| 106 | 1 | 3 | 3 | 3 | 3 | 3 | 3 | 3 | 3 | 3 | 3 | 3 | 3 | 3 | 3 | green |
| 99、185 | 1 | 1 | 1 | 3 | 3 | 3 | 3 | 3 | 3 | 3 | 3 | 3 | 3 | 3 | 3 | green |
| 147、190 | 1 | 1 | 1 | 1 | 3 | 3 | 3 | 3 | 3 | 3 | 3 | 3 | 3 | 3 | 3 | green |
| 171 | 3 | 1 | 1 | 1 | 1 | 3 | 3 | 3 | 3 | 3 | 3 | 3 | 3 | 3 | 3 | green |
| 53 | 1 | 1 | 1 | 1 | 1 | 3 | 3 | 3 | 3 | 3 | 3 | 3 | 3 | 3 | 3 | green |
| 37 | 1 | 1 | 1 | 1 | 1 | 3 | 3 | 3 | 3 | 3 | 3 | 3 | 3 | 3 | 3 | green |
| 5 | 1 | 1 | 1 | 1 | 1 | 1 | 3 | 3 | 3 | 3 | 3 | 3 | 3 | 3 | 3 | green |
| 164 | 1 | 3 | 3 | 3 | 3 | 3 | 3 | 3 | 2 | 2 | 2 | 2 | 2 | 2 | 3 | green |
| 83、84、93、139、197 | 3 | 3 | 3 | 3 | 3 | 3 | 3 | 3 | 3 | 3 | 3 | 3 | 1 | 1 | 1 | green |
| 126、128、141、150、155、166 | 3 | 3 | 3 | 3 | 3 | 3 | 3 | 3 | 3 | 3 | 3 | 3 | 3 | 3 | 1 | green |

1, 2 and 3 correspond to homozygous‘T877’/‘T877’ alleles, homozygous ‘DH1M/‘DH1Malleles and heterozygous ‘DH1M/‘T877’ alleles at the *PSH* region, respectively. SNP1, Indel1 and Indel2 located in the candidate gene.
