# Supplementary material for: Combined bulked segregant sequencing and traditional linkage analysis for identification of candidate gene for purple leaf sheath in maize
Source: PLoS One. 2018 Jan 5;13(1):e0190670. doi: 10.1371/journal.pone.0190670 (PMC5755806; doi:10.1371/journal.pone.0190670)
Supplement: S1 Fig — (a) three-leaf stage, (b) six-leaf stage, (c) T877 in adult stage, (d) DH1M in adult stage. (PDF) [file pone.0190670.s005.pdf]

(a)

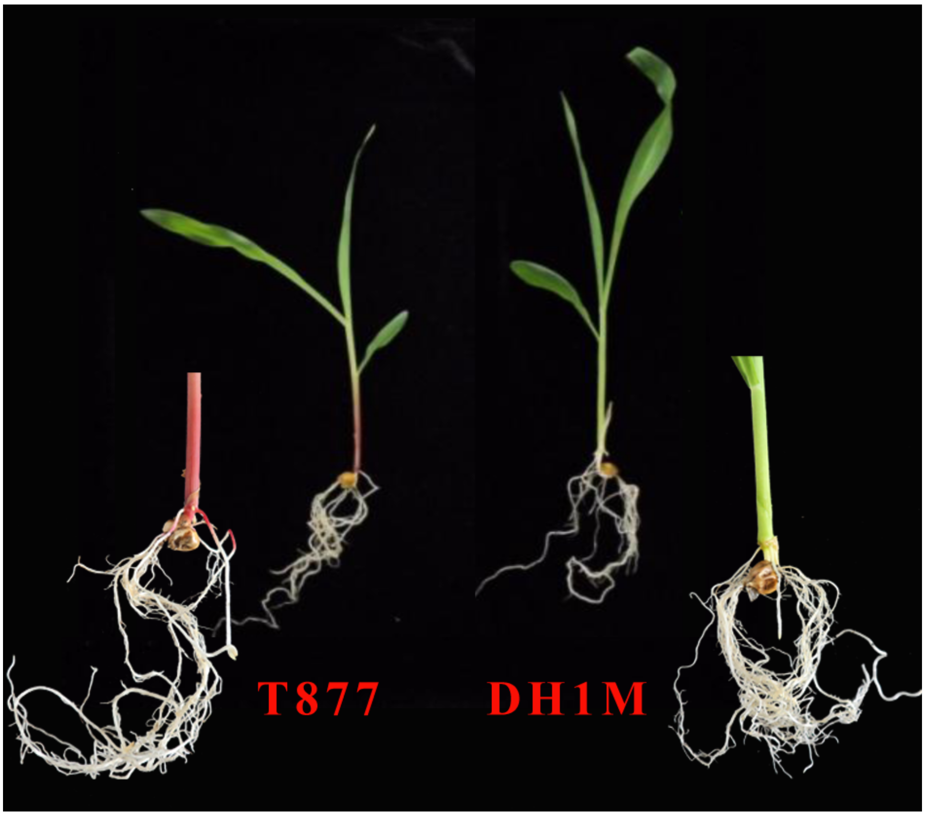

(b)

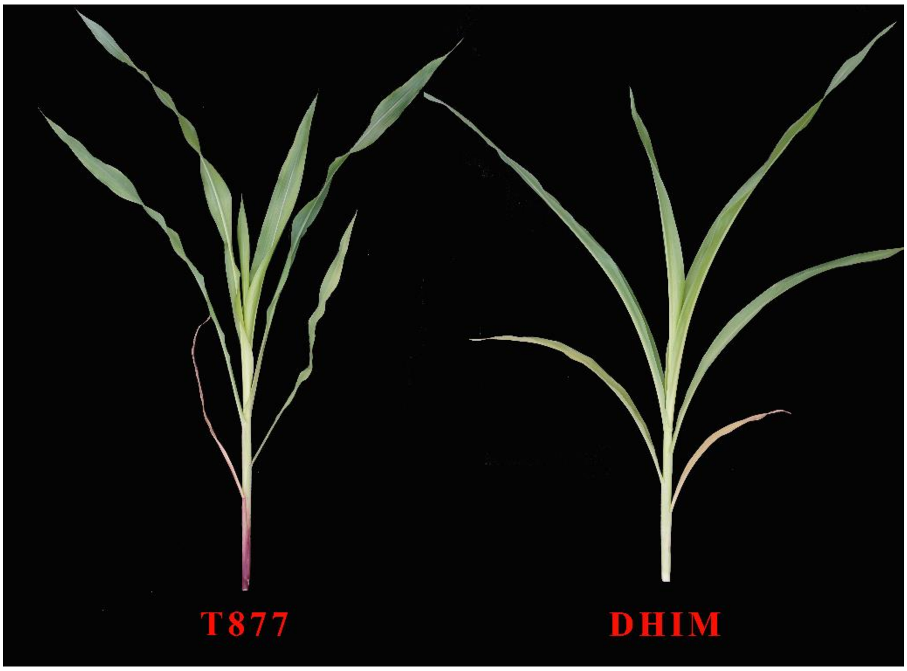

(c)

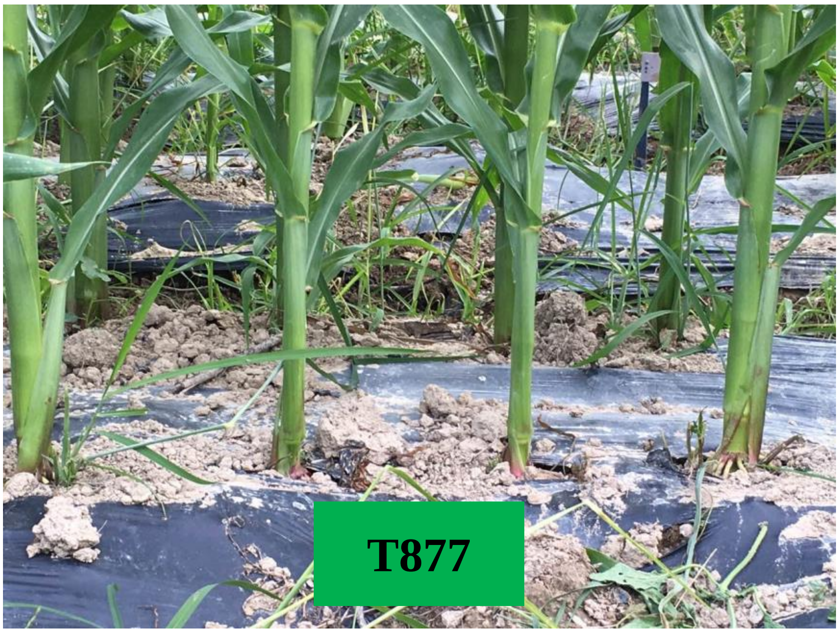

(d)

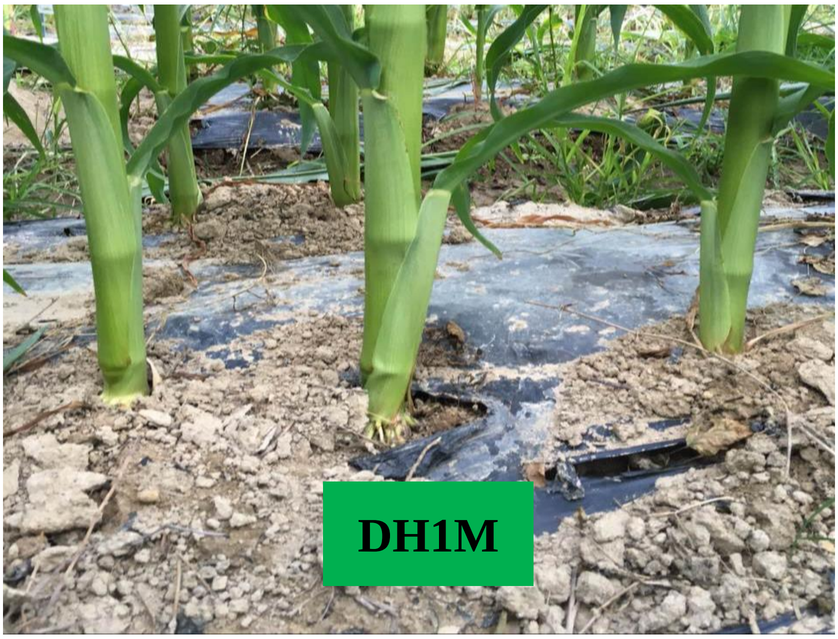

S1 Fig. Phenotypes of parental inbred lines in different stage. (a) three-leaf stage, (b) six-leaf stage, (c) T877 in adult stage, (d) DH1M in adult stage.
